# Discovery and Early Development of AVI-7537 and AVI-7288 for the Treatment of Ebola Virus and Marburg Virus Infections

**DOI:** 10.3390/v4112806

**Published:** 2012-11-06

**Authors:** Patrick L. Iversen, Travis K. Warren, Jay B. Wells, Nicole L. Garza, Dan V. Mourich, Lisa S. Welch, Rekha G. Panchal, Sina Bavari

**Affiliations:** 1 Sarepta Therapeutics, Bothell, Washington 98021, USA; Email: piversen@sareptatherapeutics.com (P.L.I.); dmourich@sareptatherapeutics.com (D.V.M); 2 United States Army Medical Research Institute of Infectious Diseases, Fort Detrick, Maryland 21702, USA; Email: Tracis.K.Warren@us.army.mil (T.K.W.); Jay.B.Wells@us.army.mil (J.B.W.); Nicole.garza@us.army.mil (N.L.G.); lisa.s.welch@us.army.mil (L.S.W.); bavaris@ncifcrf.gov (S.B.); Rekha.Panchal@us.army.mil (R.P.)

**Keywords:** Ebola, Marburg, Phosphorodiamidate Morpholino Oligomer, PMO*plus*, Therapeutic, Antiviral

## Abstract

There are no currently approved treatments for filovirus infections. In this study we report the discovery process which led to the development of antisense Phosphorodiamidate Morpholino Oligomers (PMOs) AVI-6002 (composed of AVI-7357 and AVI-7539) and AVI-6003 (composed of AVI-7287 and AVI-7288) targeting Ebola virus and Marburg virus respectively. The discovery process involved identification of optimal transcript binding sites for PMO based RNA-therapeutics followed by screening for effective viral gene target in mouse and guinea pig models utilizing adapted viral isolates. An evolution of chemical modifications were tested, beginning with simple Phosphorodiamidate Morpholino Oligomers (PMO) transitioning to cell penetrating peptide conjugated PMOs (PPMO) and ending with PMO*plus* containing a limited number of positively charged linkages in the PMO structure. The initial lead compounds were combinations of two agents targeting separate genes. In the final analysis, a single agent for treatment of each virus was selected, AVI-7537 targeting the VP24 gene of Ebola virus and AVI-7288 targeting NP of Marburg virus, and are now progressing into late stage clinical development as the optimal therapeutic candidates.

## 1. Introduction

Ebola virus (EBOV) and Marburg virus (MARV) are negative-sense, single stranded RNA viruses of the family *Filoviridae* that cause severe hemorrhagic fever resulting in high lethality. Naturally acquired human infections have occurred almost exclusively in the form of outbreaks in Equatorial Africa. These filovirus infections are characterized by acute onset of illness after an incubation period of 4 to 10 days, with initial symptoms of fever, chills, myalgia, and malaise. The disease signs and symptoms frequently become more severe including anaorexia, nausea, vomiting and abdominal pain. These are often be followed by diarrhea, respiratory complaints, conjunctival injection, hypotension, edema, prostration, confusion and coma. The hemorrhagic manifestations include coagulopathy, maculopapular rash, cytopenias, and increased transaminase levels. Most patients who are infected with Ebola virus or Marburg virus succumb to severe disease [1,2].

There are no approved treatments for filovirus infections. A recent review of various clinical trials (ClinTrials.gov June 29, 2012) revealed ongoing activities in the use of passive immunotherapy, vaccination with plasmid DNA and recombinant adenovirus vectors, and postexposure prophylaxis therapies with RNA interference (RNAi) and antisense phosphorodiamidate morpholino oligomers (PMO). Passive immunotherapy represents the first therapeutic approach to filovirus infections [3] and recent advancements employ monoclonal antibodies [4,5]. The immune therapeutic potential of eliciting anti-viral CD8+ T-cells has been demonstrated suggesting if this response were accelerated, survival will be improved [6]. The practical aspect of a vaccine approach to improve CD8+ responses which may be facilitated using plasmid DNA [7], lentiviral vectors [8], and adenovirus vectors [9] is also an active and productive area of investigation.

The search for a filovirus therapeutic has been historically challenging; however, there are examples where different approaches have demonstrated a survival benefit. A direct therapeutic approach intervening in the viral induced coagulopathy involved a recombinant nematode anticoagulant protein C2 (rNAPC2) resulted in survival of 3 of 9 treated and infected rhesus monkeys [2]. A human activated protein C (rhAPC) was also somewhat successful in providing for survival of 2 of 11 infected and treated rhesus monkeys [10]. The most robust survival benefit observed in the infected nonhuman primate lethal challenge models to date has involved viral gene-specific therapies. While significant and ongoing progress with RNAi has been reported [11], this report will focus on the use of the PMO and provide insight into the process of discovery and early stage development of PMO-based therapeutics for filoviruses.

PMOs, were designed with a number of advantages in mind [12,13] to significantly improve the stability, function, and bioavailability of antisense complexes. Most importantly, compared to conventional antisense approaches, PMOs are highly resistant to degradation [14]. PMO compounds have demonstrated reliable and effective inhibition of gene expression including c-myc [15,16,17,18,19,20], cytochrome P4503A [21,22], and viruses including vesiviruses [23,24] and Ebola virus [25]. The PMO duplex with RNA is not a substrate for RNAseH [26] and as such they can be used to induce exon skipping in genes including dystrophin [27,28] for the treatment of patients with Duchenne Muscular Dystrophy [29,30].

The PMO platform is also highly adaptable. Conjugation of the PMO compound with cell penetrating peptides (PPMO), including arginine rich peptides of up to 14 amino acids has significantly enhanced their cellular uptake leading to enhanced efficacy [31,32,33,34,35,36,37]. Peptide-conjugated PMO (PPMO) has been utilized to manipulate gene expression of numerous gram-negative bacteria including *Escherichia coli* [38,39,40,41,42], *Burkholderia cepacia complex* [43], *Acinetobacter baumanii*, and *Salmonella spp* [44,45].

The PPMO have also been successful in targeting a variety of viruses in animal challenge models including Ebola virus [46,47], coxsackievirus B3 [48], influenza A virus [49,50], alphaviruses [51], and picornaviruses [52].

Other modification such as the linkage between bases incorporating a limited number (between 2 and 5) of positive charges (PMO*plus*) also leads to enhanced efficacy and improved binding kinetics [47,53]. The PMO*plus* chemistry provides a more potent antiviral capability than the PMO and less of a limitation in tolerability than the PPMO.

## 2. Results and Discussion

The overall discovery and development strategy involved assessment of PMO drug candidates that were designed to bind to viral RNA. They were synthesized corresponding to known gene sequences of Ebola virus for L, VP24, VP30, GP, VP40, VP35, NP and flanking “leader” genome targets. Fewer sequences were prepared for Marburg virus as information gained from Ebola virus served to instruct targeting and enhanced delivery design. There were considerable attempts to optimize VP24, VP35, NP and L oligomers to increase survival rates in the murine lethal challenge model since these were most effective in the initial screening assay. Studies were also conducted utilizing in vitro translation and evaluation of infected cells in culture to improve mechanistic understanding and enhance optimization of the oligomers. If survival efficacy was observed then there was subsequent assessment in guinea pigs or non-human primates. Once these particular gene targets were associated with protection against death in the murine model, a second round of evaluation with additional PMO oligomers corresponding to the same genes but slightly different sequence were synthesized to optimize their efficacy. The strategy entailed modification of the PMO by conjugation to cell penetrating peptides (PPMO) and insertion of a limited number of modified linkages (PMO*plus*) within the oligomer was also pursued to increase efficacy or potency. Different strategies of peptide conjugation included linkage at the 3’ or 5’ end of the oligomer and differences in the amino acid sequence of the peptide. Different strategies on improving the PMO*plus* candidate included the total number of charged bases in the oligomer and selection of which base should be charged in the sequence.

### 2.1 Phosphorodiamidate Morpholino Oligomer (PMO) Chemistries Evaluated.

The phosphorodiamidate morpholino oligomer chemistries have been reported in the literature elsewhere [12,13,14,15,16,17,18,19,20,21,22,23,24,25,26,27,28,29,30,31,32,33,34,35,36,37,38,39,40,41,42,43,44,45,46,47,48,49,50,51,52,53,54,55]. A PMO, etiplersen, is currently under evaluation in clinical trials for the treatment of Duchenne Muscular Dystrophy [29,30]. The peptide conjugated PMO (PPMO) has been utilized effectively to enhance cell penetration. The PMO*plus* inserts 3 to 5 positively charged linkages into the PMO structure, which preserves stability and safety while improving cellular penetration. Each variation was interrogated for efficacy in mouse, guinea pig and nonhuman primate lethal challenge models.

**Figure 1:**
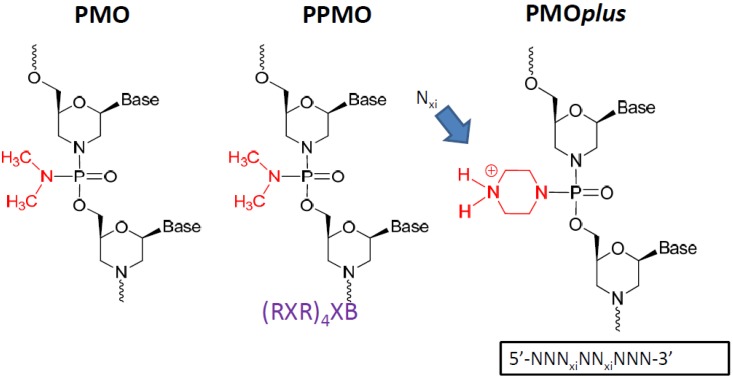
The prototype structures of the three different chemical approaches to the oligomer that were evaluated.

### 2.2 Identify Optimal Inhibitors of Viral mRNA Translation.

**Figure 2:**
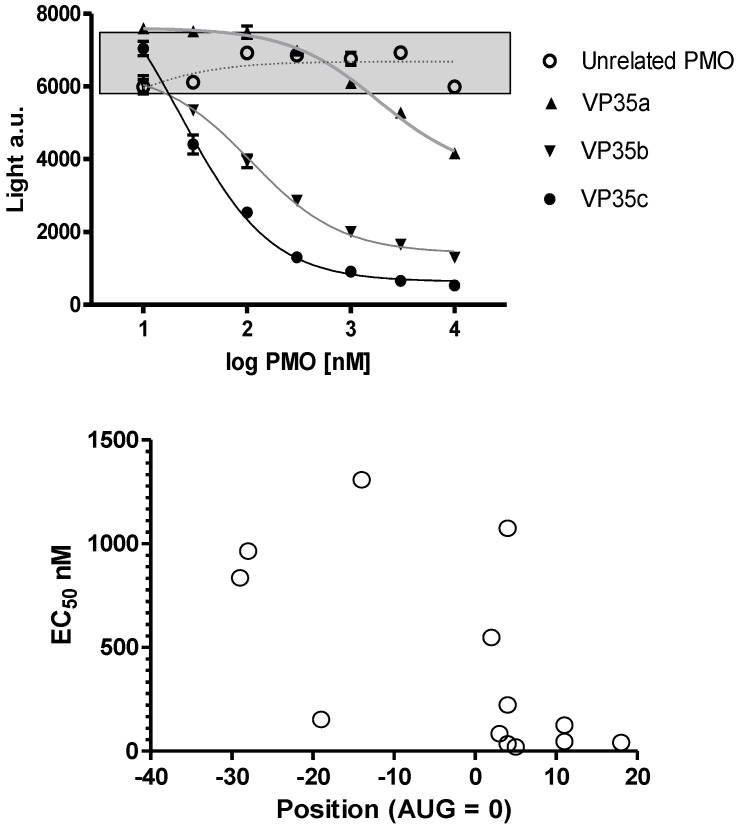
Inhibition of viral mRNA translation. (a) Three different oligomers targeting the VP35 transcript (filled symbols; VP35a = 0-1-63-413, VP35b = 0-1-63-539, and VP35c = 0-1-0-1151) and an unrelated oligomer sequence (open circles; targeting L = 0-1-63-538) incubated at different concentrations in a rabbit reticulocyte assay using a VP35 luciferase fusion gene transcript. Sigmoidal curves fit to log PMO dose versus luciferase expression revealed the potency for each oligomer sequence. All samples were evaluated in triplicate and mean and standard deviation values are presented. The grey box indicates the range of values observed in untreated control samples. (b) The EC50 for 13 different oligomers targeting VP35 (0-1-63-413, 0-1-63-539, and 0-1-0-1151), VP24 (0-1-63-537, 0-1-0-1153, NG-05-0165, and NG-05-364), L (0-1-63-538 and 0-1-0-1157) and NP (0-1-0-1147, 0-1-0-1148, 0-1-0-1149, and 0-1-0-1150) are plotted with the position of the oligomer 3’-end relative to the AUG translation start site.

The oligomer design is based on Watson-Crick base pair binding to complementary RNA sequences. The PMO are steric blockers which inhibit gene expression by physically preventing binding or progression of splicing or translational machinery components. Earlier work with the PMO compounds identified the region near the translation initiation start site as a region most likely to be effective. Based on these prior observations several PMOs targeting the Ebola virus and Marburg virus genes were designed (Table 1) and tested in a cell-free *in vitro* translation system using rabbit reticulocyte lystae (Figure 2A). Differences in inhibition potency and efficacy were observed for NP, VP24, L and VP35 viral gene transcripts. The concentration to produce a 50% reduction in translation is plotted on the ordinate and location of the 3’-end of the PMO relative to the AUG translation start site is plotted on the abscissa in Figure 2B for NP sequences 0-1-0-1147, -1148, -1149 and -1150; VP24 sequences 0-1-63-537, 0-1-0-1153, NG-05-0165, and NG-05-364; L sequences 0-1-63-538, and 0-1-0-1157; and VP35 sequences 0-1-63-413, 0-1-63-539, and 0-1-0-1151 (Table 1, Figure 2). In each case the optimal sequence extends from just below the AUG site and into the coding sequence. Overall, the in vitro translation studies indicate that the oligomers can inhibit targeted mRNA translation and support the selection of oligomers to be assessed in the mouse model.

**Table 1:** Selected Phosphorodiamidate Morpholino Oligomers (PMO) Oligomers Evaluated for Ebola virus VP35, VP24, L and nucleoprotein (NP)

Target	ID number	Sequence 5’ →3’
EBOV-NP a	0-1-0-1147	CAG ATT TTC TGA GGA CGA GAA TC
EBOV-NP b	0-1-0-1148	CAT CCA GAT TTT CTG AGG AC
EBOV-NP c	0-1-0-1149	CTC GGC GCC ATC CAG ATT TTC
EBOV-NP d	0-1-0-1150	CAT ACT CGG AAT TTT GTG ATT C
EBOV-VP35 a	0-1-63-413	GTT GTC ATC TTG TTA GAC CAG C
EBOV-VP35 b	0-1-63-539	CCT GCC CTT TGT TCT AGT TG
EBOV-VP35 c	0-1-0-1151	CCT GCC CTT TGT TCT AGT TGT C
EBOV-VP24 a	0-1-63-537	GCC ATG GTT TTT TCT CAG G
EBOV-VP24 b	0-1-0-1153	TGT ATC GTC CCG TAG CTT TAG C
EBOV-VP24 c	NG-05-0165	TTC AAC CTT GAA ACC TTG CG
EBOV-VP24 d	NG-05-364	CAG TTC AAC CTT GAA ACC TTG CG
EBOV-L a	0-1-63-538	TGG GTA TGT TGT GTA GCC AT
EBOV-L b	0-1-0-1157	GTA TTG GGT ATG TTG TGT AGC
MARV-NP	NG-05-0174	CCT AAC AAG CTA TGT AAA TC
MARV-VP35	NG-05-0264	TAT ATG ATG AGT CCC ACA TTG
MARV-VP24	NG-05-0269	CGT TGA TAA TTC TGC CAT G

### 2.3 Evaluation of Survival Benefit in the Mouse Lethal Challenge Models.

**Figure 3:**
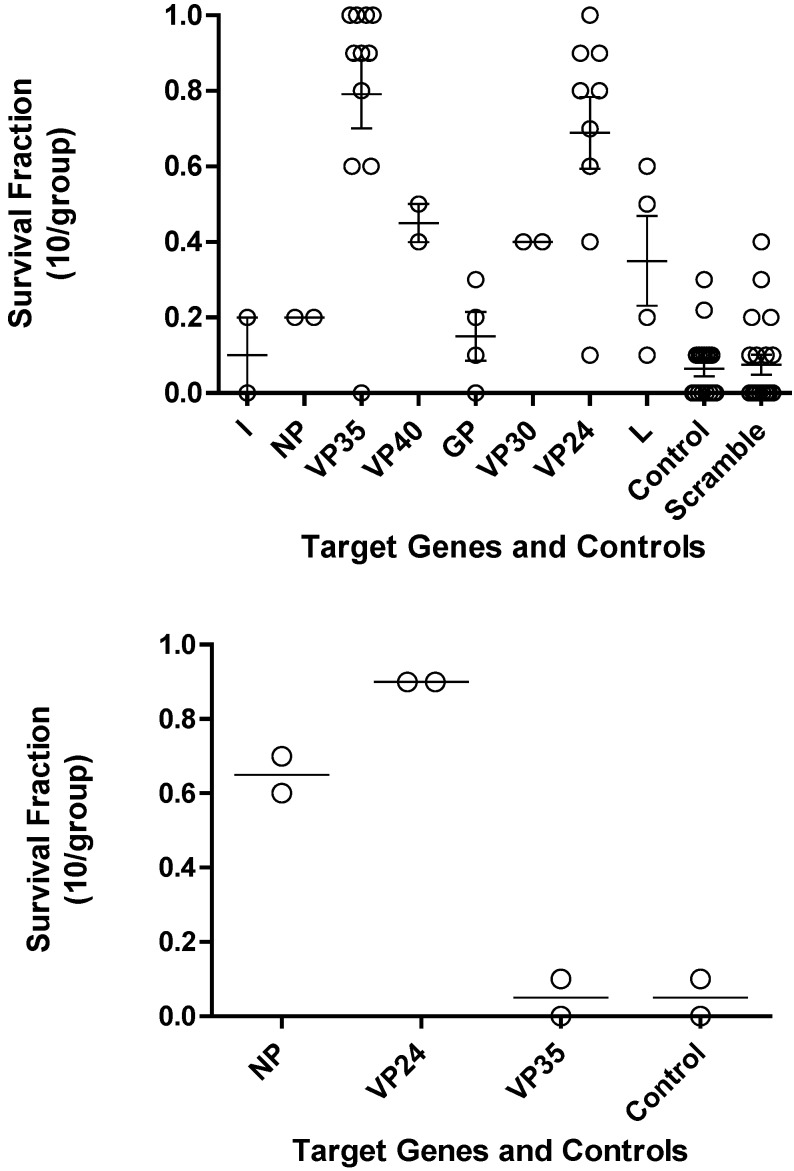
Survival fraction for PMO in Ebola virus mouse lethal challenge. (a) Each transcript of Ebola virus was investigated to identify the PMO that would produce the greatest survival benefit. Each open circle is the survival fraction (Number of survivors/Total number infected) from a separate experiment. Tukey’s multiple comparison test indicates p < 0.001 for L *vs.* VP35, GP *vs.* VP35, GP *vs.* VP24, control *vs.* VP35, control *vs.* VP24, Scramble *vs.* VP35, and Scramble *vs.* VP24; p < 0.005 for L *vs.* VP24, NP *vs.* VP35, and L *vs.* VP35; p < 0.05 for NP *vs.* VP24. (b) Bottom panel, three transcripts of mouse adapted Ravn virus (RAVV) were investigated to identify the PMO that provides the greatest survival benefit.

The optimal PMOs identified from the *in vitro* translation studies were further evaluated for their efficacy in mice. Mice were treated with either 0.5 mg of PMO oligomers targeting the different Ebola virus genes or scramble controls or vehicle controls by the intraperitoneal (i.p.) route at -24 hours and/or -4 hours prior to challenge with Ebola virus (Figure 3 Top panel and Table 2). PMO targeting VP35 and VP24 showed significantly (p<0.05) increased survival compared to the control mice. The leader, NP, VP40, and VP30 target genes were not evaluated further based on PMO survival fraction below 0.5 and their anticipated function.

The most favorable PMO that showed increased mouse survival were then further evaluated with a variety of peptides conjugated to the PMO, indicated as PPMO, and PMO*plus* as shown in Table 2. The evaluation of PPMO and PMOplus in mice was carried out at a lower dose due to their enhanced potency. Based on mouse survival data, the studies confirm that PMO, PPMO and PMO*plus* targeting Ebola virus VP35 and VP24 are as the optimal gene target candidates. The PPMO observations did not agree with the results of the PMO for GP and L targets indicating these are not reliable targets. The single GP observation was not considered further in with the PMO*plus* chemistry evaluations (Table 2).

The mouse adapted RAVV became available after multiple studies in the guinea pig had been completed. These observations guided attention to the NP target and away from GP and L. The lack of activity for VP35 was not anticipated (Figure 3, bottom panel).

**Table 2:** Different Chemical Modifications and Survival in Ebola virus Mouse Lethal Challenge Model

Viral Target	PMO (0.5 mg/mouse)*	PPMO (0.05 mg/mouse)	PMO*plus* (0.05 mg/mouse)
leader	10.0 ± 14.1 (2)	(N.T.)	(N.T.)
NP	20.0± 00.0 (2)	(N.T.)	(N.T.)
VP35	79.1 ± 30.2 (11)**	95.7 ± 7.9 (7)	68.9 ± 23.9 (9)
VP40	45.0 ±7.1 (2)	(N.T.)	(N.T.)
GP	15.0 ± 12.9 (4)	70.0 ± 00.0 (1)	(N.T.)
VP30	40.0 ± 00.0 (2)	(N.T.)	(N.T.)
VP24	68.8 ± 28.5 (9)**	63.1 ± 36.1 (61)	37.1 ± 29.6 (24)
L	35.0 ± 28.8 (4)	72.5 ± 16.4 (8)	8.3 ± 16.0 (6)
Control	6.4 ± 8.5 (19)	2.6 ± 4.6 (8)	10.0 ± 22.4 (7)
Scramble Control	7.5 ± 11.6 (20)	24.3 ± 38.1 (15)	26.7 ± 25.2 (3)

* Values represent the mean percent survival ± standard deviation and number of treatment groups in parenthesis with 10 mice per group. ** Indicates significantly different from control and scramble control, p<0.05 Tukey’s Multiple Comparison Test. N.T. Not tested.

The assessment of oligomers in the mouse lethal challenge model led to the conclusion that both VP35 and VP24 targets provided for consistent survival benefit for with PMO, PPMO and PMO*plus* oligomer chemistries. The superior potency observed for PPMO chemistry was tempered by the potential for nonspecific activity of the PPMO based on the 9.3 ratio of scramble control to the untreated controls. The concern for nonspecific activity was less for the PMO*plus* studies with the 2.7 ratio of scramble control to untreated controls.

### 2.4 Combinatorial Approach to Optimal Survival in Mice.

**Figure 4:**
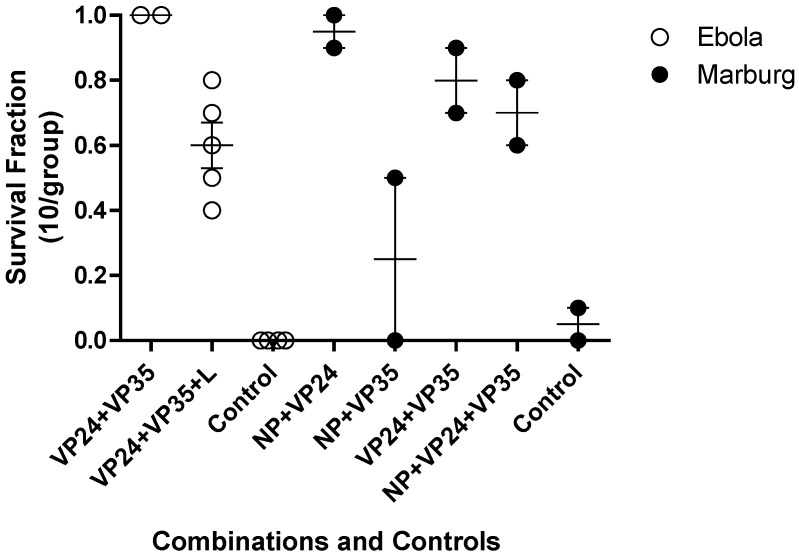
Combinations of PMO*plus* Agents in the Mouse Models. The open circles represent individual studies with mice infected with mouse adapted Ebola virus. The filled circles represent individual study groups with mice infected with mouse adapted Ravn virus (RAVV). The dose of each component oligomer was 0.05 mg/mouse so the total dose in the combination of three agents is larger than the dose for two agents.

Investigations into more than one gene target as an approach to enhanced efficacy at a relatively low dose were conducted in both Ebola and Marburg virus mouse lethal challenge models (Figure 4). This was implemented to test the hypothesis if, combination approaches might reduce the potential for emergence of viral resistance and increase the probability of therapeutic success in an outbreak setting. In each model, the combination of three agents targeting NP, VP24 and VP35 led to reduction in survival fraction relative to a combination of any two oligomers.

### 2.5 Evaluation of Survival Benefit in the Guinea Pig Lethal Challenge Models.

**Figure 5:**
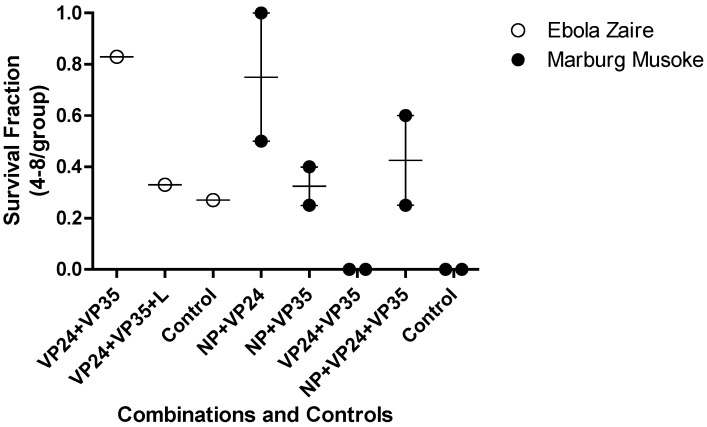
Combinations of PMO*plus* Agents in the Guine Pig Models. The open circles represent individual studies with mice infected with mouse adapted Ebola virus. The filled circles represent individual study groups with mice infected with mouse adapted Ravn virus. The dose of each component oligomer was 0.05 mg/mouse so the total dose in the combination of three agents is larger than the dose for two agents.

The guinea pig lethal challenge models have been less reliable than the mouse models in that greater survival is observed in the untreated control groups (Figure 5). The observations for Ebola tend to agree between the mouse and the guinea pig. However, the mouse RAVV agrees with guinea pig Musoke in that VP35 was not very effective (data not shown). The mouse studies indicate VP24 is superior to NP while the guinea pig indicates NP as the superior single agent (data not shown). The combination of VP35 and VP24 resulted in 80% survival in the mice infected with RAVV (Figure 4) but no survival in guinea pigs infected with Musoke (Figure 5). These observations appear to reflect a more important role for VP24 in the mice infected with RAVV than guinea pigs infected with Musoke. The combination of VP35 and VP24 was effective in Ebola Virus infected mice and guinea pigs Further, addition of a third agent targeting VP35 in the combinations diminished the survival benefit relative to the combination of NP and VP24 in both mouse mean survival for RAVV and guinea pig mean survival for Musoke from 95 to 70% and 75 to 45%, respectively. The reduced efficacy is thought to be the result of competition between the compounds for distribution to infected tissues suggesting caution in adding less effective agents to a combination. The two studies in guinea pig with the PMO*plus* agents indicate the combination of NP with VP24 provided the greatest survival benefit. The studies in mouse agree with those studies in guinea pig in the optimal survival benefit from the NP combined with VP24 (Figure 5).

A summary of guinea pig data is provided in Table 3, which reveals the challenge due to survival in the untreated control groups. No significant differences were observed between controls and treatment groups. The maximum survival was observed with PMO*plus* in a two combination treatment group targeting Ebola virus VP 35 (AVI-7539) with 5 PMO*plus* linkages within the 20-mer with the following sequence CC+T GCC C+TT TGT +TCT +AGT +TG and VP 24 (AVI-7537) with 5 PMO*plus* linkages within the 19-mer with the following sequence GCC +ATG GT+T TT+T TC+T C+AG G. This combination is referred to as AVI-6002.

**Table 3:** Survival in Ebola virus Guinea Pig Lethal Challenge Model

Target	PMO		PPMO		PMO*plus*	
	Dose (mg/kg)	Percent Fraction	Dose (mg/kg)	Percent Survival	Dose (mg/kg)	Percent Survival
**VP35**	20	25 ± 12 (2)	-	-	20	38 ± 00 (1)
**VP24**	-	-	-	-	20	49 ± 12 (2)
**L**	-	-	-	-	20	13 ± 00 (1)
**VP35 + VP24**	-	-	-	-	20	83 ± 00 (1)
**VP35 + VP24 + L**	20	46 ± 26 (4)	1	12 ± 18 (2)	20	33 ± 00 (1)
**Control**	-	33 ± 47 (4)	-	25 ± 35 (2)	-	27 ± 18 (1)

The values are mean percent survival ± standard deviation and numbers in parenthesis are the number of replicate studies.

### 2.6 Evaluation of Survival Benefit in the Nonhuman Primate Lethal Challenge Models.

**Figure 6:**
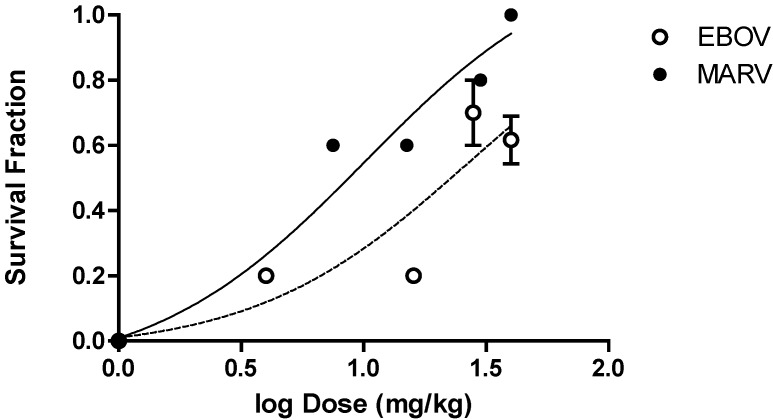
Dose-Dependent Survival Benefit. The filled circles represent survival fraction (Number survivors/Number Infected) versus log dose (mg/kg) for Cynomolgus macaques infected with Marburg virus and treated with different doses of AVI-6003 (targeting VP24 and NP). The open circles represent the survival fraction versus log dose (mg/kg) for Rhesus macaques infected with Ebola virus and treated with AVI-6002 (targeting VP35 and VP24).

The PMO*plus* oligomer chemistry resulted in survival benefit that was dose dependent in nonhuman primates infected with either Ebola virus or Marburg virus. Observations from four independent studies with Ebola virus and four independent studies with Marburg virus were combined to generate a dose versus survival curve shown in Figure 6. The estimated dose required to result in 50% survival was 27 mg/kg for AVI-6002 and 10 mg/kg for AVI-6003. The maximal observed survival for AVI-6002, targeting VP35 (AVI-7539) and VP24 (AVI-7537), was 8 of 13 (2/4, 3/4, and 3/5) or 61% at 40 mg/kg. The maximal survival for AVI-6003, targeting VP24 (AVI-7287) and NP (AVI-7288), was 13 of 13 (9/9 and 4/4) or 100% at 40 mg/kg. The target genes are different for the two agents, the infected nonhuman primate genus is different and the oligomer composition is different confounds interpretation of why AVI-6003 appears to be more potent and effective than AVI-6002.

The summary in Table 4 provides a comparison of the three types of chemistry (PMO from 4 different studies; PPMO from a single study; and PMO*plus* from two studies) and confirms the PMO*plus* provides the greatest survival benefit.

**Table 4:** Chemistry Comparison in Ebola virus Nonhuman Primate Lethal Challenge Model.

Target	PMO		PPMO		PMO*plus*	
	Dose (mg/kg)	Percent Survival^*^	Dose (mg/kg)	Percent Survival	Dose (mg/kg)	Percent Survival
VP35	300	00 (0/4)	-	-	-	-
(AVI-7539)**						
VP35+VP24	-	-	-	-	40	61 (8/13)
(AVI-6002)**						
VP35 + VP24 + L	350-450^***^	25 (3/12)	18 (MTD)	00 (0/3)	-	-
(AVI-7539, AVI-7537, NG-06-0648)**						
Control	-	00 (0/4)	-	00 (0/1)	-	00 (0/2)

* Percent survival, these numbers of survivors over the total number treated are indicated in parenthesis. Long-term survival was determined by those animals that survived to 28 days post infection. ** The numbers refer to the PMOplus but the sequences are the same as for the PMO and PPMO *** Two studies are combined in which the dose ranged from 350 to 450 mg/kg/

**Figure 7:**
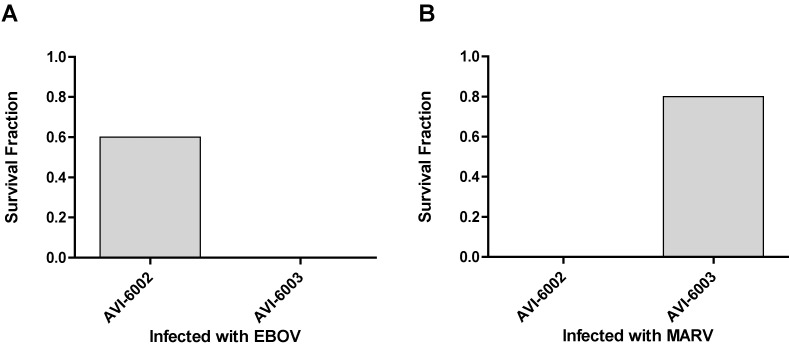
Specificity of AVI-6002 and AVI-6003 for Ebola and Marburg viruses. (a) Rhesus macaques infected with Ebola virus were treated *i.v.* with 40 mg/kg AVI-6002 or 40 mg/kg AVI-6003. (b) Cynomolgus macaques infected with Marburg virus were treated *i.v.* with 30 mg/kg AVI-6002 or 30 mg/kg AVI-6003.

To evaluate specificity, the studies indicated in Figure 7 were conducted. Nonhuman primates were infected with Ebola virus and a group (N=5) was treated with AVI-6002 resulted in 3 of 5 survivors indicating a sequence specific effects survival benefit while the other group (N=4) was treated with AVI-6003 resulted in 0 of 4 survivors indicating no evidence of sequence nonspecific survival benefit (Figure 7A). Conversely, Cynomolgus macaques were infected with Marburg virus and 0 of 4 treated with AVI-6002 survived and 4 of 5 treated with AVI-6003 survived (Figure 7B). 

**Figure 8:**
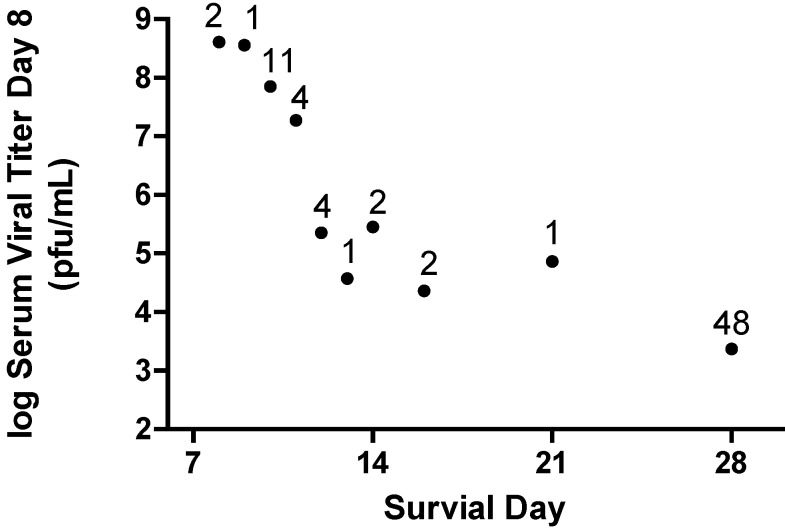
Reduction in Viremia on Day 8 Post Infection Predicts Survival. Data are included for 76 Cynomolgus macaques infected with 1000 pfu of Marburg virus by the subcutaneous route. Each symbol represents the geometric mean viral titer for animals that survive to the indicated day. The number above the symbol indicates the number of animals for the observation. The survivors indicated at 28 days include animals that are long-term survivors with some surviving up to 40 days.

The design of AVI-6002 and AVI-6003 was based on inhibition of viral gene expression of genes considered essential for viral growth and replication. Reduction in Marburg virus viral titer is reliably observed in nonhuman primates treated with the AVI-6003 (Figure 8). These observations tend to confirm the design strategy is successful. However, a significant aspect of the viral pathogenesis involves host response to viral antigens and cellular damage caused by the viral infection. The data provided in Figure 8 show the log_10_ serum viral titer on day eight post challenge from all individual nonhuman primates infected with Marburg virus (N=76) and the survival outcome for those individuals. A relationship is established in which the lower the serum viral titer on day 8, the greater the potential for extending survival time.

**Figure 9:**
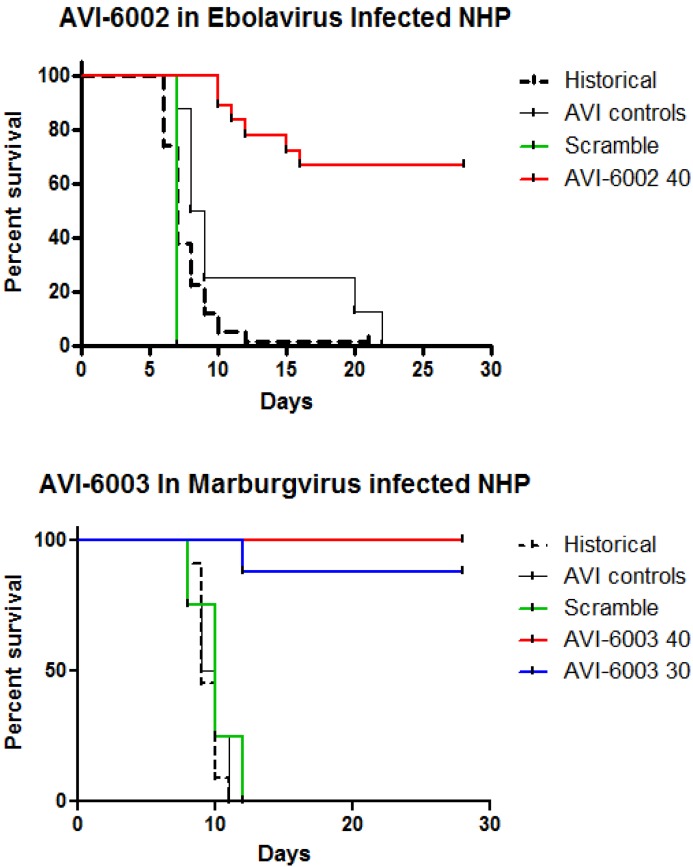
Combined Nonhuman Primate Survival Observations. (a) AVI-6002, a combination of AVI-7537 and AVI-7539, was utilized to evaluate the survival benefit. The observations include 58 historical controls, 8 controls, 4 scramble controls and 18 treated Rhesus macaques infected with Ebola Zaire. (b) AVI-6003, a combination of AVI-7287 and AVI-7288, was utilized to evaluate survival benefit. The observations include 11 historical controls, 4 AVI controls, 4 scramble sequence controls and 8 treated Cynomolgus macaques infected with Marurg Musoke.

AVI-6002 is effective in treating Ebola virus in the nonhuman primate lethal challenge model (Figure 9; p < 0.0001). In addition, AVI-6002 effectively reduces plasma viral titer and diminishes multiple aspects of viral induced pathology (data not shown). In a parallel manner, AVI-6003 is effective in treating nonhuman primates infected with Marburg virus (Figure 8B; p < 0.0001). AVI-6003 also reduces plasma viral titer and diminishes multiple aspects of viral induced pathology (data not shown). Both of these agents have been administered as single intravenous injections to healthy volunteers with no resulting serious adverse events.

Survival benefit and viral titer reduction has been established for AVI‑6002, which consists of two oligomers, AVI-7537, which targets Ebola virus VP24 mRNA, and AVI‑7539, which targets VP35 mRNA. However, a critical element in moving toward studies to evaluate safety in humans is the need to evaluate the least complex dose regimen and the lowest dose. Two important questions related to overall safety of AVI‑6002 are whether (1) both components of AVI‑6002 are necessary for efficacy and (2) both components of AVI-6002 are necessary for preventing the development of resistant virus.

The two oligomer components of AVI-6002 (AVI‑7537 and AVI‑7539) were recently evaluated. The long-term survival from AVI‑7537 targeting VP24 mRNA is greater than that observed in the AVI-6002 treatment group and no survival benefit was observed from AVI-7539 alone in this study. The data indicates AVI‑7537 is the dominant contributor to efficacy observed with AVI‑6002 and is now the single agent for treatment of Ebola virus (data not shown).

In a parallel fashion, survival benefit and viral titer reduction has been established for AVI-6003, composed of AVI-7287, which targets VP24 mRNA, and AVI-7288, which targets NP mRNA. Studies have recently been conducted which demonstrate AVI-7288 provides the survival benefit and reduction in viral titer that is observed in AVI-6003 and no survival benefit or reduction in viral titer is observed from AVI-7287. The data indicate AVI-7288 is the dominant contributor to the efficacy observed with AVI-6003 and is now the single agent for treatment of Marburg virus (data not shown).

The characteristics of AVI-7537 and AVI-7288 are described in Table 5. Studies are in progress at this time to evaluate the potential for viral genome mutations that may result in viral resistance, establish the relationship between pharmacokinetics and efficacy, and the potential efficacy at increasing times after viral exposure. These studies as well as other late stage development efforts will provide the basis for establishing the human effective dose, which is essential for the intended animal rule approval.

**Table 5:** Physical Characteristics of AVI-7357 for Ebola virus and AVI-7288 for Marburg virus.

	AVI-7537	AVI-7288
Sequence	GCC +ATG GT+T TT+T TC+T C+AG G	GAA TAT TAA C+AI +AC+T GAC +A+AG TC
Melting Temperature	81.0°C	84.2°C
Molecular Weight	6826 grams/mole	8179 grams/mole
Percent GC	47.4 %	30.4%
Target and Position	VP24 10330	NP 75
Association constant	9.97 X 10^7^ M^-1^s^-1^	7.3 X10^6^ M^-1^s^-1^
Dissociation constant	1.30 X 10^-5^ s^-1^	4.72 X10^-5 ^s^-1^
Equilibrium Binding	1.30 X 10^-13^ M	6.47 X 10^-12^ M
EC_50_ (inhibit translation)	585 nM	872 nM

## 3. Experimental Section

### 3.1 Oligomer synthesis and purification.

The PMO oligomers were synthesized by AVI BioPharma (Corvallis, Oregon; now Sarepta Therapeutics in Cambridge, MA) as described by Summerton and Weller [1]. The spectrum of PPMO oligomers were described in Swenson et al., 2007 [2] and were prepared by AVI BioPharma. The synthesis of the cell penetrating peptides was described by Wu et al., 2007 [3]. The PMOplus oligomers were described in Warren et al., 2010 [4] and were synthesized by AVI BioPharma. 

### 3.2 In Vitro Translation.

The protein coding sequence for firefly luciferase, without the initiator-Met codon AUG, was subcloned into the multiple cloning site of the plasmid pCiNeo. Synthetic oligodeoxynucleotides were designed to provide the 5’-UTR through the translation start site of EBOV VP35 (-98 to +39; bases 3020-3157), EBOV VP24 (-84 to +43; bases 10261-10390), EBOV L (-80 to +49; bases 11501-11632) and EBOV NP (-89 to +40) were duplexed and subcloned into the Nhe 1 and Sal 1 sites. The single AUG in each viral encoded protein is in frame with the coding sequence for firefly luciferase. The plasmids were linearized with Not I, and *in vitro* transcribed RNA was produced using the T7 polymerase-based Megascript kit and protocol (Ambion, Austin, Texas). *In vitro* translations using a rabbit reticulocyte lysate (Promega) were carried out by mixing different concentrations of PMO, PPMO or PMO*plus* with transcribed RNA at a final concentration between 40pM to 5nM. Luciferase light emission was read on an FLx800 microplate luminometer (BioTek Instruments, Inc., Winooski, VT).

### 3.3 Mouse Lethal Challenge Models.

A series of lethal murine-adapted Ebola virus (MA-EBOV) challenge studies were performed at USAMRIID in C57 BL6 mice. A total of 237 different PMO, PPMO and PMOplus compounds were evaluated over three years. The purpose of these studies was to screen PMO Ebola virus drug product concepts either as a single agent or in combination for their ability to protect C57 BL6 mice (average weight of 20 grams) from death after a lethal MA-EBOV challenge. Mice were challenged with 1,000 pfu (200 µL total volume) of MA-EBOV stock by intraperitoneal injection (IP) [Bray P1 (14Oct05), titer: 1 x10^5^ pfu/mL]. The usual route of administering the study drug or combination of study drugs was by an IP route at -24 hours and/or -4 hours prior to IP lethal viral challenge. A total of 4040 mice were divided into 404 study groups of 10 mice/group with 1820 involved in evaluation of PMO compounds, 1440 for PPMO compounds and 780 for PMO*plus* compounds. Survival outcome was assessed after Day 14 post challenge. All PMO treated mice failures died between 6 and 14 days after viral challenge so there was no obvious delay in death compared to the untreated control mice.

Mice in groups of 10 were challenged with 1,000 pfu (200 µL total volume) of the mouse-adapted Ravn virus (RAVV) viral stock by intraperitoneal injection [(15Mar07) m(25)PP4(3X)V(1), titer: 7.5 x107 pfu/mL]. A separate experiment utilized Marburg virus (MARV) Ci67 which appears to be less completely mouse adapted based on the 50% survival in the control group. One hour prior to viral challenge, PMO*plus* drugs were administered by the intraperitoneal route at 20mg/kg per drug as single agents, 2-agent combination, or a 3-agent combination to the mice. One challenge group of untreated mice served as a control in each experiment. Control animals showed classic signs of illness beginning at Day 6. By Day 8, most control mice die. Survival outcome was assessed at the end of the study on Day 14.

### 3.4 Guinea Pig Lethal Challenge Models.

A series of PMO, PPMO or PMO*plus* drugs targeting VP24, VP35, and L Ebola virus gene targets were assessed in the lethal guinea pig model studies at USAMRIID. A total of 234 guinea pigs were divided into 42 study groups of 4-6 guinea pig/group with 82 involved in evaluation of PMO compounds, 16 for PPMO compounds and 146 for PMO*plus* compounds. In this model, the guinea pig-adapted Ebola virus isolate (EBOV-Mayinga) when administered at a 1,000 pfu, should cause death in challenged Hartley guinea pigs between 7 and 14 days following IP injection. Under our test conditions, all study drugs were administered via an intraperitoneal injection beginning at +1 h. Survival outcome was assessed at Day 21 in the guinea pig model.

Guinea pigs (average weight of ~0.5 kg) were challenged via intraperitoneal injection with a freshly thawed and diluted aliquot of a 1,000 pfu [MARV-Musoke MV6 GP8 pp#3 (3X) E6(2), 15Mar96, titer (1x108 pfu/mL)]. One group that did not receive any administration except the 1,000 pfu viral challenge served as the control for each the challenge experiment. Guinea pigs were administered study drug via an IP route at different dosage levels and specific time points. The number of guinea pigs per treatment group varied from 4 to 8 animals. Interpretation of results was possible if all control animals died in each experiment. Survival outcome was assessed on Day 21 after viral challenge. Untreated control guinea pigs die between 7 and 14 days after lethal viral challenge.

### 3.5 Nonhuman Primate Lethal Challenge Studies.

A series of PMO, PPMO or PMO*plus* drugs targeting VP24, VP35, and L Ebola Zaire gene targets were assessed in lethal nonhuman primate model studies at USAMRIID. The lethal challenge of 1,000 pfu is considered sufficient to predict exposure that would occur in humans after accidental exposure or intense exposure in the field. The route of viral challenge was via an intramuscular route as opposed to an intraperitoneal route employed in EBOV murine and guinea pig lethal challenge models The time until death is expected to be observed between 7 and 12 days.

### 3.6 Statistical Analysis.

Animals were assigned to treatment groups using varying degrees of randomization with mouse and guinea pig studies relying on cage assignments of groups and formal procedures to ensure randomized animal assignments to treatment groups for nonhuman primate studies. Mouse and guinea pig studies were not blinded but nonhuman primate studies were conducted using single-blinded procedures such that the animal handlers were not informed of the identity of treatment groups. Survival rates from replicate experiments utilized a Fisher exact test with stepdown bootstrap adjustment and survival curves were evaluated by a Log-rank (Mantel-Cox) test using Prism GraphPad Software (GraphPad Inc, LaJolla, CA.)

## 4. Conclusions

Sarepta Therapeutics has developed a proprietary adaptable platform chemistry, phosphorodiamidate morpholino oligomers (PMO) containing up to five positive linkages (PMO*plus*), that have significantly improved the stability, efficacy, specificity, delivery, and safety of antisense complexes. Two new therapeutic agents have been identified, AVI-7537 targeting the VP24 transcript of Ebola virus and AVI-7288 targeting the NP transcript of Marburg virus. These novel agents have been carefully evaluated in a battery of Good Laboratory Practuce (GLP) toxicology and safety pharmacology studies, they have open investigational new drug (IND) applications, and have been evaluated in early phase I clinical trials.

AVI-7537 is a PMOplus oligomer that binds directly to the viral VP24 transcript RNA is the optimal single agent for protection against a Ebola Zaire lethal challenge. The VP24 protein is an inhibitor of type I interferon responses. Thus inhibition of VP24 may lead to a more efficient host response to infection. VP24 also forms homodimers and binds to VP35 as well as NP and may play a role in the switch from viral replication to transcription, a function critical to the viral life cycle. This may explain the mechanism of action of AVI-7537.

AVI-7288 is a PMO*plus* olligomer that binds directly to viral RNA of the nucleoprotein (NP) transcript with a binding equilibrium constant of between 6.5 10^-12^M. The Marburg virus particle is composed of 7 structural proteins. Four of them, NP, VP35, VP30 and L, form the nucleocapsid complex that surrounds the viral genome. The nucleocapsid protein, NP, is detected in 2 forms in infected cells (92 and 94 kDa). The 94 kDa form, a phosphorylated protein, appears to interact with VP35, which binds to the RNA-dependent RNA polymerase L and is essential for transcription and replication of the viral RNA genome. The non-phosphorylated, 92 kDa, form of NP is thought to bind strongly to viral RNA leading to encapsidation of viral RNA. Phosphorylation of NP occurs in region II, amino acids 439 – 475, of the C-terminal portion of NP occurs in an overlapping region with the RNA binding site of the protein, amino acids 289 – 352. Phosphorylation of NP leads to weaker binding of NP to RNA, due to repulsion of negatively charged RNA to the negative phosphate groups, resulting in limited access of the polymerase complex to the RNA template. These data suggest NP is a molecular switch which in the phosphorylated form facilitates transcription and replication necessary for the early portion of the viral life-cycle and a non-phosphorylated form that serves to encapsidate the viral RNA genome in preparation for viral egress from an infected cell. AVI-7288 is designed to inhibit NP synthesis resulting in catastrophic effects on production of viral mRNA, viral genome replication, and viral assembly.

The studies to date have identified NP and VP24 as the key antiviral targets for Marburg virus and Ebola virus, respectively. Mutations in NP and VP24 appear to be required to mouse adapt these viruses so that virulence is observed. Further, VP24 mutations are required for Ebola virus to replicate in the guinea pig. The mutations resulting in mouse adaption are not identical to those required for replication in the guinea pig suggesting an explanation for efficacy of the VP24/VP35 combination in mice and the lack of efficacy in the guinea pig.

Observations from *in vitro* translation observations are surprisingly informative. The assay removes entanglement of cellular penetration and potential chaos in signal from tens of thousands of non-targeted cellular molecules. The VP35a PMO failure (Figure 2A) to completely inhibit translation points to unappreciated complexity in the translation process. Conversely, the capacity of VP35c to completely inhibit translation provides insight into the importance of the optimal target position in the transcript. The importance of location is confirmed by multiple studies with different transcripts (Figure 2B) leading to a conclusion that steric blocking PMOs that bind to a position around +10 relative to the translation start site will be more potent. This observation has been repeated for dozens of eukaryotic and viral transcripts. A secondary conclusion is that the Tm (thermal melt temperature) of the oligomer is not as important as the targeting position. In addition, the optimal length of a PMO designed to inhibit translation is between 20 and 22 nucleobases [39,54,55]. These features of target position in the transcript and optimal length facilitate the process of discovery to screening for effective gene targets and enable “Rapid Response” in the creation of medical countermeasures for biothreat and emerging infectious diseases. Several exercises have been executed to explore the potential of the PMO platform technology for rapid response (Table 6).

**Table 6:** Rapid Response Drug Discovery with the PMO Platform Technology.

Challenge	Response	Outcome
12 Aug 2002: Feline Calicivirus	Lethal outbreaks in Atlanta, GA and Eugene, OR	47/50 treated kittens survive
		3/31 untreated kittens survive [24]
15 Oct 2002: West Nile Virus	Lethal outbreak in penguins at Milwaukee Zoo. Concept to compound in 7 days.	3/3 treated penguins survive
		1/8 untreated penguins survive
		IND filed in 2003, Phase Ib
11 Feb 2004: Ebola virus	Accident at USAMRIID. Concept to delivery in 7 days.	Effective in mouse, guinea pig and nonhuman primate. IND filed. Phase I studies
5 June 2009: Pandemic Influenza A virus	TMT*-Rapid Response 1. Concept to compound in 7 days	Effective in mouse and ferret. IND filed in 2010. Phase I initiated
5 Oct 2010: Dengue 2 virus	TMT-Rapid Response 2. Concept to compound in 10 days	Efficacy in mouse and ferret models
21 March 2011: Two undisclosed agents	TMT Rapid Response 3. Concept to compounds for both pathogens in 18 days	Compounds placed in storage for future testing

*Translational Medical Technologies

Screening PMOs targeting viral gene targets in the mouse and guinea pig lethal challenge models encompasses all of the traditional strengths and weaknesses identified by drug development for countless therapeutics. This discovery process utilized both the mouse and guinea pig and evaluated two related but distinct viruses. Interesting differences emerged early in the discovery process in that VP35 was an important target in Ebola virus but not in Marburg virus (Figure 3) and the combination of VP24 and VP35 was very effective for Ravn virus infected mouse model but not effective in the Marburg Musoke infected guinea pig (Figure 4 and Figure 5). However, confidence was enhanced by the reproducible efficacy of the VP35 and VP24 combination for Ebola in the mouse (Figure 4) guinea pig (Figure 5) and nonhuman primate (Table 4). Similar observations were observed for the NP and VP24 combination for Ravn virus and Marburg virus. The operating hypothesis during screening in these models was that the evolution of these viruses would lead to elimination of less important genes and all targets are likely to be equally active. The observations clearly lead to the conclusion that inhibition of the expression of some viral genes is more important than others (Figure 3). Differences in transcript target abundance, accessibility of the viral RNA target, the half-life of the resulting expressed viral protein, and subcellular localization of the viral product may explain why some genes are less effective when targeted by the PMO. The optimal viral gene target is likely to be critical for viral growth and its role in interaction with the host, particularly the immune response.

**Figure 10:**
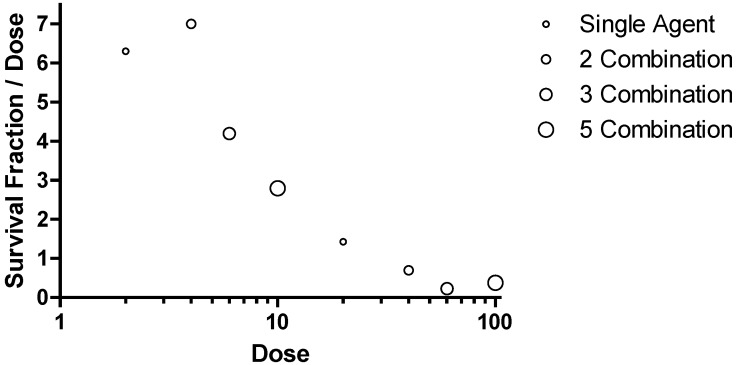
Combination Survival Benefit in the Ebola virus Mouse Lethal Challenge Model. Data were plotted with the survival fraction divided by the total dose on the ordinate and the total dose administered is plotted on the abscissa.

The development strategy to identify two gene targets for each virus was based on a lack of confidence in the sequence conservation of viral genome in the PMO target region. If is important to ensure antiviral activity in the setting of a viral quasispecies. Further, a combination of two viral targets provided potential flexibility and insurance of efficacy. However, the addition of multiple components presented a potential liability that a less effective agent may compromise the activity of an active component. This was observed for Ebola virus in that addition of the L target to the VP24 and VP35 combination resulted in diminished efficacy in the mouse model (Figure 4). This was also observed in the Marburg virus discovery when adding VP35 to the VP24 and NP combination in both the mouse (Figure 4) and guinea pig (Figure 5). This led to a broader examination of the combination data from the Ebola mouse lethal challenge model in which more extensive combinations were evaluated (Figure 10).

This method of presenting data would represent a horizontal line if adding additional targets resulted in equivalent benefit, a trend towards positive slope if adding additional targets showed synergy in providing survival benefit and a negative sloping trend if adding additional targets to a combination showed antagonism in providing survival benefit. The data for comparison at 2 and 20 mg/kg for each component agent is included in the figure for single agents, combination of two agents (2 Combination), combination of 3 agents (3 Combination) and the combination of five agents (5 Combination). The symbols on in the figure are circles with increasing diameter to help identify the increasing number of agents in a combination. The survival observations for combinations of 3 and 5 all provide a high degree of survival but require a commensurate greater dose. The peak or optimal survival for a given dose of PMO is with a combination of two agents at a dose of 2 mg/kg each (Figure 10). These observations support the conclusion that fewer components are desired from a functional point of view but also from an added risk from multiple agents in the later stage development. The optimal therapeutics are the single agents AVI-7537 targeting the VP24 gene of Ebola virus and AVI-7288 targeting the NP gene of Marburg virus.

The overall strategy and platform characteristics reported here provide the template for identifying therapeutics for other emerging infectious disease. Once the developmental template has been refined, this strategy has the potential to efficiently discover and develop new medical countermeasures to biothreat agents.
